# Deubiquitination and stabilization of EIF4A3 by OTUB2 contributes to TPI1-mediated glycolysis and TNBC progression

**DOI:** 10.1186/s13058-026-02260-5

**Published:** 2026-03-19

**Authors:** Zhiyong Wang, Xiao Chen, Xiao Han, Xuchen Cao, Xin Wang

**Affiliations:** 1 The First Department of Breast Cancer, Tianjin Medical University Cancer Institute and Hospital, National Clinical Research Center for Cancer, Huan Hu Xi Road, He Xi District, Tianjin, 300060 People’s Republic of China; 2 Key Laboratory of Breast Cancer Prevention and Therapy, Tianjin Medical University, Ministry of Education, Tianjin, 300060 People’s Republic of China; 3 Key Laboratory of Cancer Prevention and Therapy, Tianjin, 300060 People’s Republic of China; 4 Tianjin’s Clinical Research Center for Cancer, Tianjin, 300060 People’s Republic of China; 5 Department of General Surgery, The Affiliated Hospital of Inner Mongolia Medical University, Hohhot, 010050 Inner Mongolia Autonomous Region People’s Republic of China

**Keywords:** Triple-negative breast cancer (TNBC), Proliferation, Invasion, OTUB2, EIF4A3, TPI1

## Abstract

**Background:**

Tumor cells rely on enhanced glycolytic capacity to facilitate cell growth and progression. However, the molecular mechanisms governing glycolysis in triple-negative breast cancer (TNBC) remain largely elusive. In this study, we elucidate the role of OTUB2 in regulating glycolysis in TNBC and delve into the mechanisms.

**Methods:**

We utilized The Cancer Genome Atlas (TCGA) dataset to analyze the expressions of OTUB2, EIF4A3, and TPI1 in breast cancer tissues and specifically in triple-negative breast cancer (TNBC) tissues. qPCR and western blot experiments were performed to confirm the protein expressions of OTUB2, EIF4A3, and TPI1 in TNBC tissues and cell lines. Subsequently, overexpression and knockdown experiments followed by cell functional assays were conducted to elucidate the impact of OTUB2 on EIF4A3/TPI1-mediated TNBC malignant progression and related molecular mechanisms. Finally, a xenograft mouse model was established to verify the effect of OTUB2 in vivo.

**Results:**

TCGA database indicated highly expressions of OTUB2, EIF4A3, and TPI1 in breast cancer tissues and TNBC tissues. Consistently, the mRNA and protein expressions of OTUB2, EIF4A3, and TPI1 were increased in TNBC tissues and cell lines, as compared to para-carcinoma tissues and MCF10A cells. OTUB2 knockdown inhibited cell proliferation, migration and invasion, and decreased glucose uptake, ATP level, lactate production, and ECAR, while the OTUB2 overexpression exhibited the opposite trends. Further data elucidated that OTUB2 stabilized the EIF4A3 protein via deubiquitination, and EIF4A3 interacted with TPI1 to stabilize its mRNA. Overexpression of EIF4A3 exacerbated TNBC malignant activities and glycolysis, while silencing TPI1 reversed these effects. In vivo, the knockdown of OTUB2 significantly inhibited tumor growth in a xenograft mouse model.

**Conclusions:**

We conclude that OTUB2 deubiquitinates and stabilizes EIF4A3 to promote TNBC progression via TPI1-mediated glycolysis of tumor cells.

**Supplementary Information:**

The online version contains supplementary material available at 10.1186/s13058-026-02260-5.

## Introduction

Breast cancer, a prevalent type of malignant tumor affecting women, exhibits high incidence and mortality rates globally [1]. Triple-negative breast cancer (TNBC), characterized by the absence of estrogen receptor (ER), progesterone receptor (PR), and human epidermal growth factor receptor-2 (HER-2) expression, comprises approximately 15–20% of breast cancers [2]. TNBC exhibits a highly aggressive clinical course, often affecting younger individuals, with a high propensity for metastasis and a grim prognosis [3]. Hence, there is a pressing need to further explore the mechanisms underlying TNBC progression and identify potential therapeutic targets.

Deubiquitinases (DUBs), proteases within the ubiquitin-proteasome system, are responsible for cleaving ubiquitin from substrates and disassembling polyubiquitin chains [4]. The ovarian tumor domain (OTU) deubiquitinylating cysteine proteases, specifically OTUB1 and OTUB2 (OTU ubiquitin aldehyde binding 1 and 2), are exemplary members of the OTU subfamily within the deubiquitinylase enzyme [5, 6]. Researches have demonstrated that OTUB2 plays a role in promoting the development of various cancers, including breast cancer [7], non-small cell lung cancer [8], endometrial cancer [9], and liver cancer [10], et al. However, there is a scarcity of studies focusing on TNBC, and the molecular mechanisms concerning the role of OTUB2 in TNBC remain unclear. The Cancer Genome Atlas (TCGA) analysis revealed high expression level of OTUB2 in TNBC tissues, so it is speculated that OTUB2 may contribute to TNBC progression by mediating the deubiquitination of downstream proteins.

Eukaryotic initiation factor 4 A-III (EIF4A3) serves as a pivotal component of the exon junction complex and plays crucial roles in mRNA splicing, localization, transport, and translation [11]. As an RNA binding protein (RBP), EIF4A3 enhances the expression of downstream genes by binding to mRNA transcripts in upstream regions [12]. TCGA analysis in this study showed that EIF4A3 expression was significantly elevated in TNBC tissues. Previous studies have reported that EIF4A3 promotes breast cancer progression by stabilizing downstream circRNAs [13, 14]. However, the precise downstream molecular mechanisms of EIF4A3 remain to be fully elucidated. The bioinformatic predictions have identified multiple ubiquitination sites in EIF4A3, leading to the hypothesis that overexpression of OTUB2 might be involved in EIF4A3 deubiquitination, thereby stabilizing its expression.

Triosephosphate isomerase 1 (TPI1) is a key glycolytic enzyme that holds a pivotal part in the glucose metabolic pathway and participates in a wide array of biological functions [15]. TPI1 encodes a triphosphate isomerase, composed of two identical subunits, which catalyzes the mutual conversion of glyceraldehyde-3-phosphate and dihydroxyacetone phosphate in the glycolysis and gluconeogenesis pathways, respectively [16]. Our TCGA analysis revealed high expression levels of TPI1 in TNBC tissues. Furthermore, existing research has demonstrated that TPI1 activates the PI3K/AKT/mTOR signaling pathway, promotes breast cancer progression by stabilizing CDCA5, and enhances tumor cell glycolysis [17]. At present, there are few studies exploring the effect of TPI1 on breast cancer, and the underlying mechanisms remains unclear. The stareBase predictions suggest that EIF4A3 can bind to TPI1 mRNA, so we hypothesize that EIF4A3 may stabilize TPI1 mRNA and thereby promote its expression.

In the current study, we propose the hypothesis that OTUB2 deubiquitinates and stabilizes EIF4A3, which in turn leads to the stabilization of TPI1 mRNA, ultimately contributing to glycolysis and TNBC progression. Our findings are expected to provide useful therapeutic and diagnostic targets for TNBC.

## Materials and methods

### Clinical data

The expressions of OTUB2, EIF4A3, and TPI1 were analyzed based on data collected from breast cancer patients and specifically those with TNBC, sourced from The Cancer Genome Atlas (TCGA) dataset. Additionally, 30 paired TNBC tissues and para-carcinoma tissues were obtained from TNBC patients who underwent surgical resection at Tianjin Medical University Cancer Institute and Hospital between January 2022 and August 2024. All participating TNBC patients had not undergone radiotherapy or chemotherapy prior to surgery. Prior information and written consent were secured from all participants prior to initiating the study. This research was granted approval by the Ethics Committee of Tianjin Medical University Cancer Institute and Hospital (approval no.8247311).

### Cell culture and treatment

Five human TNBC cell lines were studied: MDA-MB-231, MDA-MB-453, MDA-MB-468, SUM-159, and BT-549. MDA-MB-231, MDA-MB-453 and MDA-MB-468 cells were obtained from ATCC (Rockville, MD, USA). SUM-159 and BT-549 cells were originally obtained from Asterand, Plc. (Detroit, MI, USA). MCF10A mammary epithelial cells were purchased from ATCC. MDA-MB-231, MDA-MB-453, MDA-MB-468, and BT-549 cells were grown in RPMI-1640 medium (Sigma-Aldrich, St. Louis, MO, USA). MCF10A cells were cultured in DMEM/F-12 supplemented with insulin (0.01 mg/mL), epidermal growth factor (20 ng/mL) and hydrocortisone (500 ng/mL). SUM-159 cell lines were cultured in DMEM/F-12 supplemented with insulin (5 µg/mL). All media were supplemented with 10% heat-inactivated fetal calf serum, 2 mM L-glutamine, 100 U/mL penicillin and 100 µg/mL streptomycin (Sigma Aldrich). All the cell were incubated at 37 °C in humidified 5% CO_2_, 95% air atmosphere.

To inhibit mRNA synthesis in MDA-MB-468 cells, 2 µg/mL actinomycin D (Sigma Aldrich) was added to cells followed by an incubation for 0, 3, 6, and 9 h. Relative gene mRNA remaining was then measured.

### lentivirus infection

The lentivirus carrying OTUB2 short hairpin RNAs#1/2/3 (sh-OTUB2#1/2/3), control shRNAs (sh-NC), EIF4A3 shRNAs (sh-EIF4A3), TPI1 shRNAs (sh-TPI1), OTUB2 overexpression vector (oe-OTUB2), EIF4A3 overexpression vector (oe-EIF4A3), TPI1 overexpression vector (oe-TPI1), and control vector (oe-NC) were all procured from GenePharma (Shanghai, China). For cell infection, the final viral titer was 1 × 10^8^ TU/mL and 20 µL lentivirus was added into 1 mL medium to infect cells. Stable cell lines were selected with puromycin (Sigma, 2 µg/mL) for 2 weeks following standard protocols [18].

### Quantitative PCR (qPCR)

Total RNA was extracted from cells or tissues utilizing Trizol reagents (TaKaRa, Tokyo, Japan). Subsequently, the RNA was reverse-transcribed into cDNA. PCR amplification was carried out using SYBR Green qPCR Master Mix (Thermo Fisher, Waltham, MA, USA), adhering to the manufacturer’s guidelines, on a 7900HT Fast Real-Time PCR machine (Applied Biosystems). The relative gene expressions were normalized to the internal reference gene GAPDH and calculated using the 2^−ΔΔCt^ method. PCR reaction procedure: Initial pre-denaturation at 95 °C for 30 seconds, performed once; followed by 40 cycles of denaturation at 95 °C for 5 seconds and annealing/extension at 60 °C for 30 seconds. The primer sequences utilized are provided below: OTUB2, F: 5′-TGCACTCACGAAGTAGAGCC-3′, R: 5′-TGAAGAGCCGGAATGTTCCAT-3′; EIF4A3, F: 5′-GATGCCGATGAACGTTGCTG-3′, R: 5′-GGTGGTGGCACCTTAGAAGTAT-3′; TPI1, F: 5′- CCCAGGAAGTACACGAGAAG-3′, R: 5′- CAGTCACAGAGCCTCCATAAA-3′; GAPDH, F: 5′- TTCCGTGTCCCCACTGCCAACGT − 3′, R: 5′- CAAAGGTGGAGGAGTGGGTGTCGC − 3′.

### Western blot analysis

Total proteins from cells and tissues were extracted with RIPA lysis buffer (Sigma-Aldrich) on ice, and total protein was extracted for following SDS-PAGE analysis. The primary antibody information was as follows: The primary antibodies used were as follows: anti-OTUB2 (1:1000, ab74371, Abcam, UK), anti-EIF4A3 (1:500, ab308024, Abcam, UK), anti-TPI1 (10713-1-AP, Proteintech, Wuhan, China), and anti-GAPDH (1:1000, ab8245, Abcam, UK). The secondary antibody was anti-rabbit IgG (1:3000, ab205718, Abcam, UK) and anti-mouse IgG (1:3000, ab6728, Abcam, UK). Finally, the images of western blot were visualized with an enhanced chemiluminiscent (ECL) method.

### Cell counting kit-8 (CCK-8) assay

We utilized a CCK-8 kit (CK04, Dojindo, Shanghai, China) to assess the proliferation of MDA-MB-468 and BT-549 cells. Specifically, 2 × 10^4^ cells were seeded in each well in a 96-well plate, with a total volume of 100 µL per well in medium supplemented with 10% FBS. Subsequently, a working solution was prepared by adding 10 µL of CCK-8 reagent to 90 µL of medium, and 100 µL of this solution was dispensed into each well. The cells were then incubated for 2 h. Following this, the absorbance at 490 nm was measured using a microplate reader (Thermo Fisher, USA).

### 5-ethynyl-2′-deoxyuridine (EdU) assay

MDA-MB-468 and/or BT-549 cells were cultivated in a 96-well plate and exposed to 100 µL of medium containing 20 µM EdU (CA1170, Solarbio, Beijing, China). Following an incubation period at 37 °C with 5% CO_2_ for 2 h, the cells underwent fixation with 4% paraformaldehyde and permeabilization with 0.5% Triton X-100. Subsequently, nuclei were counterstained with DAPI (C0060, Solarbio, Beijing, China). The outcomes were captured using a fluorescence microscope (Olympus, Tokyo, Japan). For analysis, images were taken from five randomly selected areas.

### Colony formation assay

Cells were trypsinized and plated at a density of 500 cells per 6 cm dish, with each dish containing 10 mL of culture medium. The cells were then allowed to grow for an additional 2 weeks at 37 °C, 5% CO_2_, and 100% humidity, with no medium changes during this period. Afterward, the colonies were rinsed with PBS, fixed using 4% paraformaldehyde, and stained with crystal violet for a duration of 5 min. Image J software was utilized to quantify the number of colonies.

### Transwell assay

For the migration assay, 600 µL of RPMI-1640 supplemented with 10% FBS was placed in the lower chamber of a transwell plate (8 μm pore size, BD Biosciences, USA). Simultaneously, 200 µL of RPMI-1640 containing 5 × 10^4^ cells were added to the top chamber. After incubating for 24 h, the cells remaining on the upper surface of the membrane were gently removed using a cotton swab. Subsequently, the cells that had migrated to the lower surface of the membrane were fixed with 4% paraformaldehyde and stained with crystal violet. For the invasion assay, the transwell chamber was coated with matrigel matrix (BD Biosciences) prior to use. Then, 600 µL of RPMI-1640 containing 10% FBS was added to the lower chamber, and 200 µL of RPMI-1640 containing 5 × 10^4^ cells were placed in the upper chamber. After 24 h of incubation, the cells on the lower surface of the membrane were fixed and stained as described for the migration assay. The number of migrating or invading cells was counted using a light microscope (Olympus).

### Metabolic assay

After specific lentivirus infections, glucose uptake and lactate production in MDA-MB-468 and/or BT-549 cells were tested using the glucose uptake colorimetric assay kit (K676-100, Biovision, USA) and lactate colorimetric assay kit (K627-100, Biovision, USA), and ATP production was detected via an ATP Assay Kit (S0026, Beyotime, Shanghai, China) following the manufacturers’ protocols [19].

### Extracellular acidification rate (ECAR) detection

Glycolysis was assessed by measuring the ECAR using the Seahorse XFe 96 Extracellular Flux Analyzer (Seahorse Bioscience, USA) as previous description [20]. Briefly, approximately 2 × 10^4^ cells were plated into a cell culture microplate compatible with the Seahorse XFe 96 system. Following a baseline measurement, glucose (10 mmol/L), oligomycin (2 µmol/L), and the glycolysis inhibitor 2-deoxy-glucose (50 mmol/L) were sequentially injected into each well at specified time points. The obtained data were analyzed using the Seahorse XFe 96 Wave software and presented in units of mPH/min.

### Co-immunoprecipitation (Co-IP)

MDA-MB-468 cells were lysed using cell lysis buffer (Beyotime). Following clarification, an aliquot of the whole cell lysates was set as the input control. The remaining cell lysates were incubated with a primary antibody anti-OTUB2 (5 µg/mL, H00078990-M14, Thermo Fisher) overnight at 4 °C. Subsequently, Protein A&G beads (Invitrogen) were added and incubated for an additional 4 h at 4 °C. Following this, the beads were washed thoroughly and eluted using SDS-loading buffer at 95 °C for a duration of 5 min. The eluted proteins were then subjected to identification through western blot analysis.

### Cycloheximide (CHX) assay

After specific infection with sh-OTUB2 or sh-NC, MDA-MB-468 cells were processed with 50 µg/mL CHX (66-81-9, TargetMol, USA) treatment for 0, 2, 4, 6, and 8 h. Then, western blot analysis was performed to measure EIF4A3 protein expression to judge the half-life of cells.

### Ubiquitination detection

The stability of EIF4A3 in MDA-MB-468 cells upon OTUB2 silencing was investigated through in vivo ubiquitination assays. Specifically, cells from the control, sh-OTUB2, and oe-OTUB2 groups were treated with 10 µM MG132 (133407-82-6, MedChemExpress, USA) for a duration of 8 h. Subsequently, the ubiquitination level of EIF4A3 was assessed by IP using an anti-EIF4A3 antibody, followed by western blot analysis employing an anti-Ub antibody (1:1000, 10201-2-AP, Proteintech).

### In vitro deubiquitination assay

Ubiquitinated EIF4A3 (Ub-V5/EIF4A3-Flag) was first purified from 293T cell lysates using anti-Flag M2 Affinity Gel (Sigma-Aldrich). The protein complexes containing HA tagged wild-type OTUB2 or the C51S mutant type of OTUB2 were purified from 293T cells using anti-HA Affinity Gel (Sigma-Alrich). For the in vitro deubiquitination assay, the purified substrate was incubated with either purified wild-type or mutant OTUB2 for 1 h at 37 °C in reaction buffer. Subsequently, EIF4A3-Flag was purified and analyzed by immunoblotting with antibodies against V5 to assess ubiquitination levels.

### RNA immunoprecipitation (RIP)

The RIP assay was performed using the Magna RIP Kit (MAGNARIP02, Millipore, USA). Briefly, 293T cells or MDA-MB-468 cells were lysed with RIP lysis buffer. Subsequently, the lysates were incubated in RIP buffer containing magnetic beads that were conjugated with anti-IgG (2 µg/mL, sc-515946, Santa cruz, USA) or anti-Ago2 antibody (1:100, MABE253, Millipore, Germany) for a period of 4 h. Following this incubation, the RNA was extracted from the magnetic beads using TRIzol reagent. The enrichment of TPI1 was then quantified using qPCR.

### RNA pull-down assay

293T cells or MDA-MB-468 cells were transfected with biotinylated (bio)-TPI1 or a control. After being washed with PBS, the cells were lysed using the specific lysis buffer. The lysate was then incubated with M-280 streptavidin magnetic beads (Sigma, USA) for a duration of 3 h. Following this, the RNA bound to the beads was extracted using Trizol reagent. The protein level of EIF4A3 was subsequently evaluated by performing a western blot analysis.

### Xenograft models

Female BALB/c nude mice (*N* = 20, aged 6–8 weeks, weighing 20 ~ 25 g) were acquired from Hunan SJA Laboratory Animal Co., Ltd. (Changsha, China). All research protocols were approved by the Institutional Animal Care and Ethical Standards Committee of Tianjin Medical University Cancer Institute and Hospital (approval no.8247311). The mice were then transplanted with stable sh-OTUB2-expressing cells, stable oe-OTUB2-expressing cells, or the control cells (5 × 10^6^ cells in 100 µL) into right thoracic mammary fat pads of mice. The animals were divided into four groups, each containing five mice: sh-NC, sh-OTUB2, oe-NC, and oe-OTUB2. Tumor sizes were measured every seven days using a vernier caliper, and the tumor volumes were calculated according to the formula: tumor volume = (length × width²) / 2. On day 35, the mice were anesthetized and euthanized, and the tumors were carefully excised for weighing and further analysis.

### Immunohistochemical staining

Tumor tissues from nude mice were fixed, paraffin-embedded, and cut into 4 μm sections. Then, sections were applied to immunohistochemical staining using anti-Ki-67 (1:500, ab320709, Abcam), anti-OTUB2 (1:100, PA5-99680, Invitrogen), anti-EIF4A3 (1:100, MA5-41263, Invitrogen), and anti-TPI1 (1:200, MA5-18291), respectively. Five representative fields were counted, and the positive staining rate was presented.

### Statistical analysis

SPSS version 25.0 (IBM, Armonk, YK, USA) was used for statistical analysis. All data are expressed as means ± SD. Two-group comparisons were analyzed by an unpaired Student’s t test (two-tailed). Differences between the means of multiple groups were compared by one-way ANOVA followed by Tukey’s multiple-comparisons test. Statistical significance was considered at *P* < 0.05.

## Results

### Elevated OTUB2 expression in TNBC tissues and cell lines

**Fig. 1:**
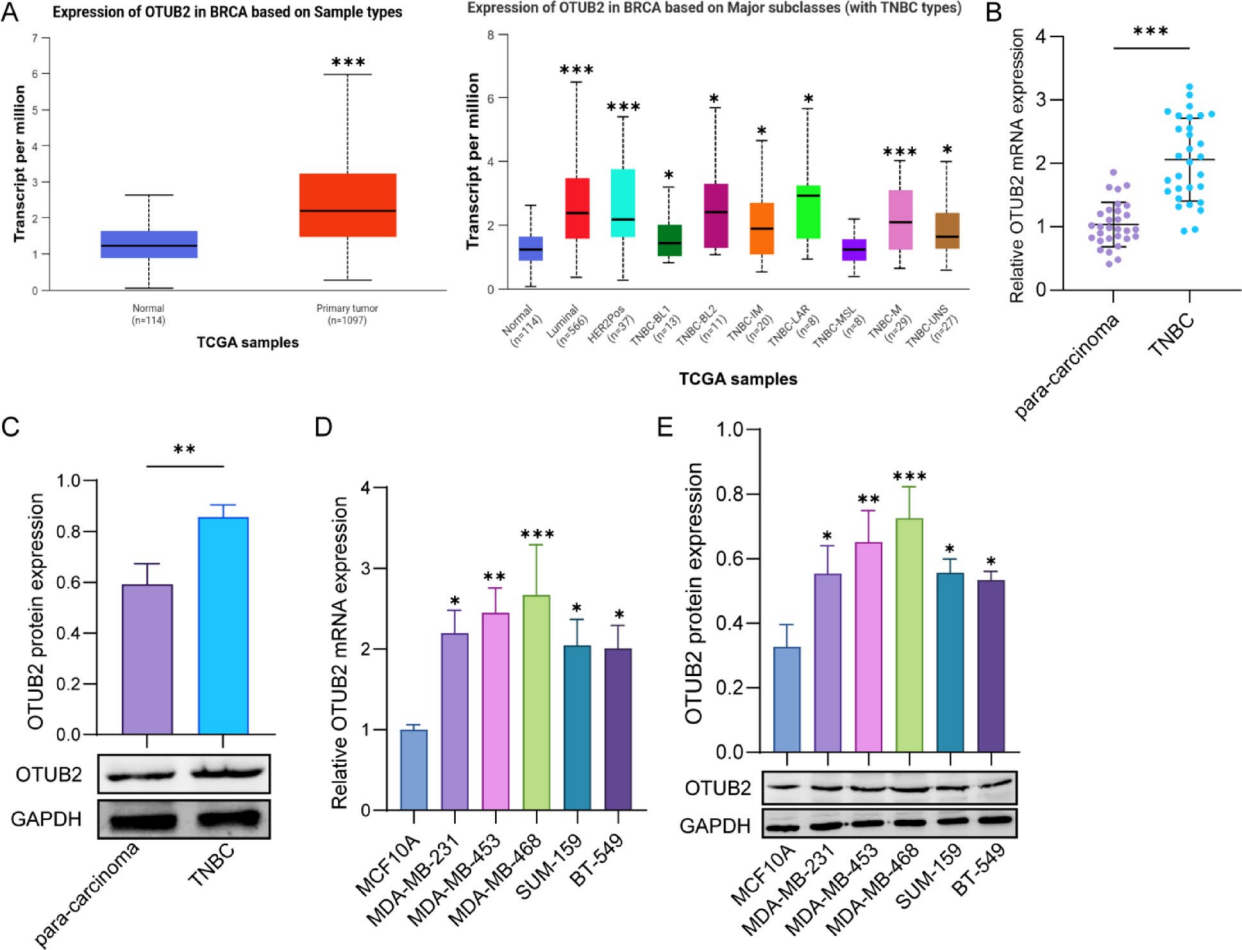
Elevated OTUB2 expression in TNBC tissues and cell lines. **A** The Cancer Genome Atlas (TCGA) database was used to analyze OTUB2 expression in breast cancer tissues and TNBC tissues. *p < 0.05; ***p < 0.001. **B** and **C** qPCR and western blot detected the expression of OTUB2 in TNBC tissues and para-carcinoma tissues (*n* = 30 samples per group). **p < 0.01;***p < 0.001. **D** and **E** qPCR and western blot detected the expression of OTUB2 in MCF10A, MDA-MB-231, MDA-MB-453, MDA-MB-468, SUM-159, and BT-549 cells (*n* = 3 per group). *p < 0.05; **p < 0.01; ***p < 0.001. Results are expressed as mean ± SD. Statistical analyses were analyzed using a Student’s t-test in A (left), B, and C, while one-way ANOVA followed by Tukey’s multiple-comparisons test were used to analyze differences between groups in A (right), D, and E

Based on the clinical data from TCGA database, OTUB2 expression was elevated in breast cancer tissues and TNBC tissues (Fig. 1A). Then, the qPCR and western blot showed that OTUB2 mRNA and protein expressions were highly expressed in TNBC tissues, as compared to the para-carcinoma tissues (Fig. 1B and C). Subsequently, it was found that the expression levels of OTUB2 mRNA and protein were elevated in TNBC cell lines when compared to MCF10A cells, and the MDA-MB-468 cells exhibiting the highest OTUB2 expression and BT-549 cells with the lowest OTUB2 expression were chosen for further experiments (Fig. 1D and E). These data indicated the elevated OTUB2 expression in TNBC tissues and cell lines.

### OTUB2 facilitates the malignant activities of TNBC cells

**Fig. 2:**
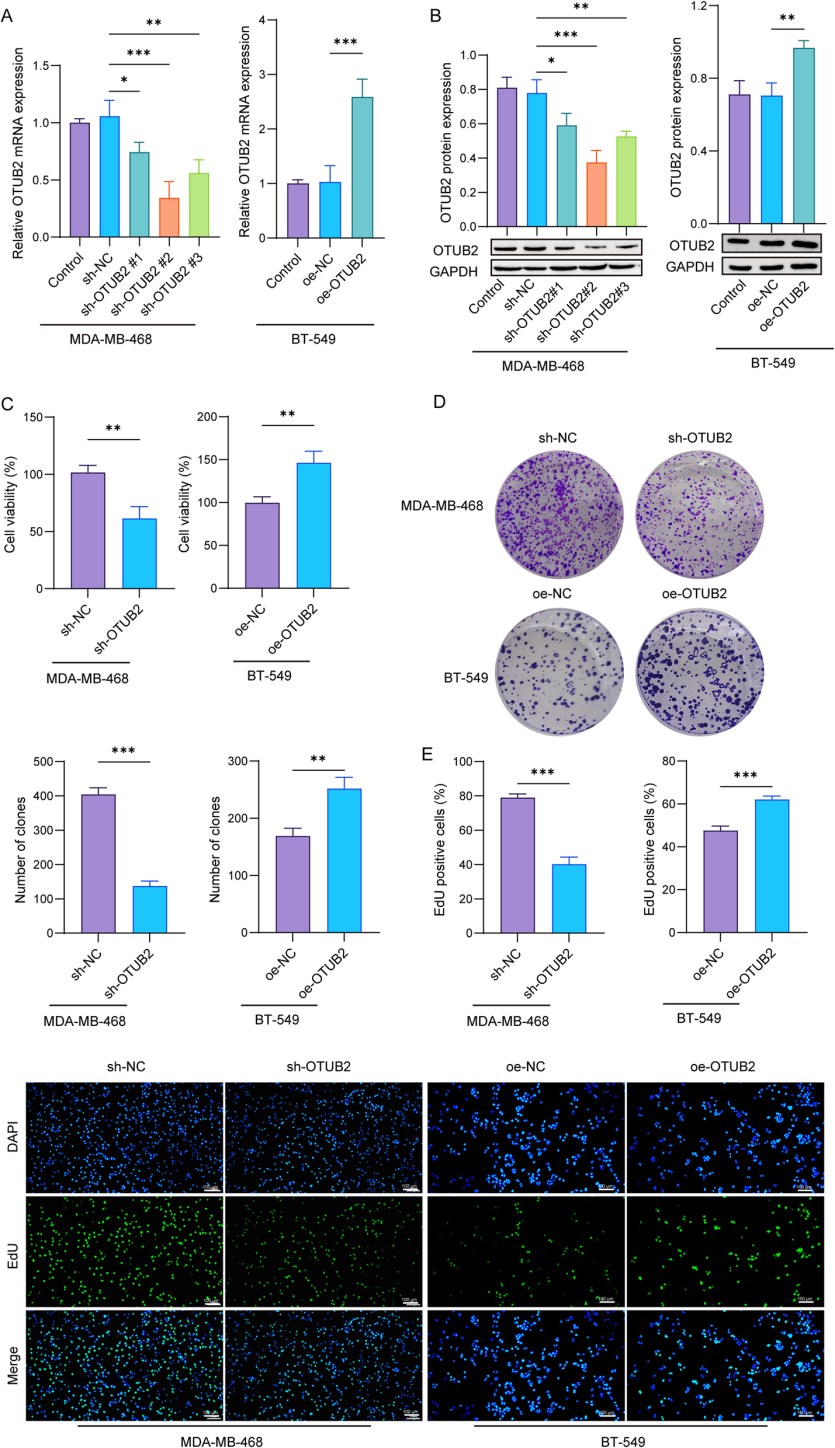

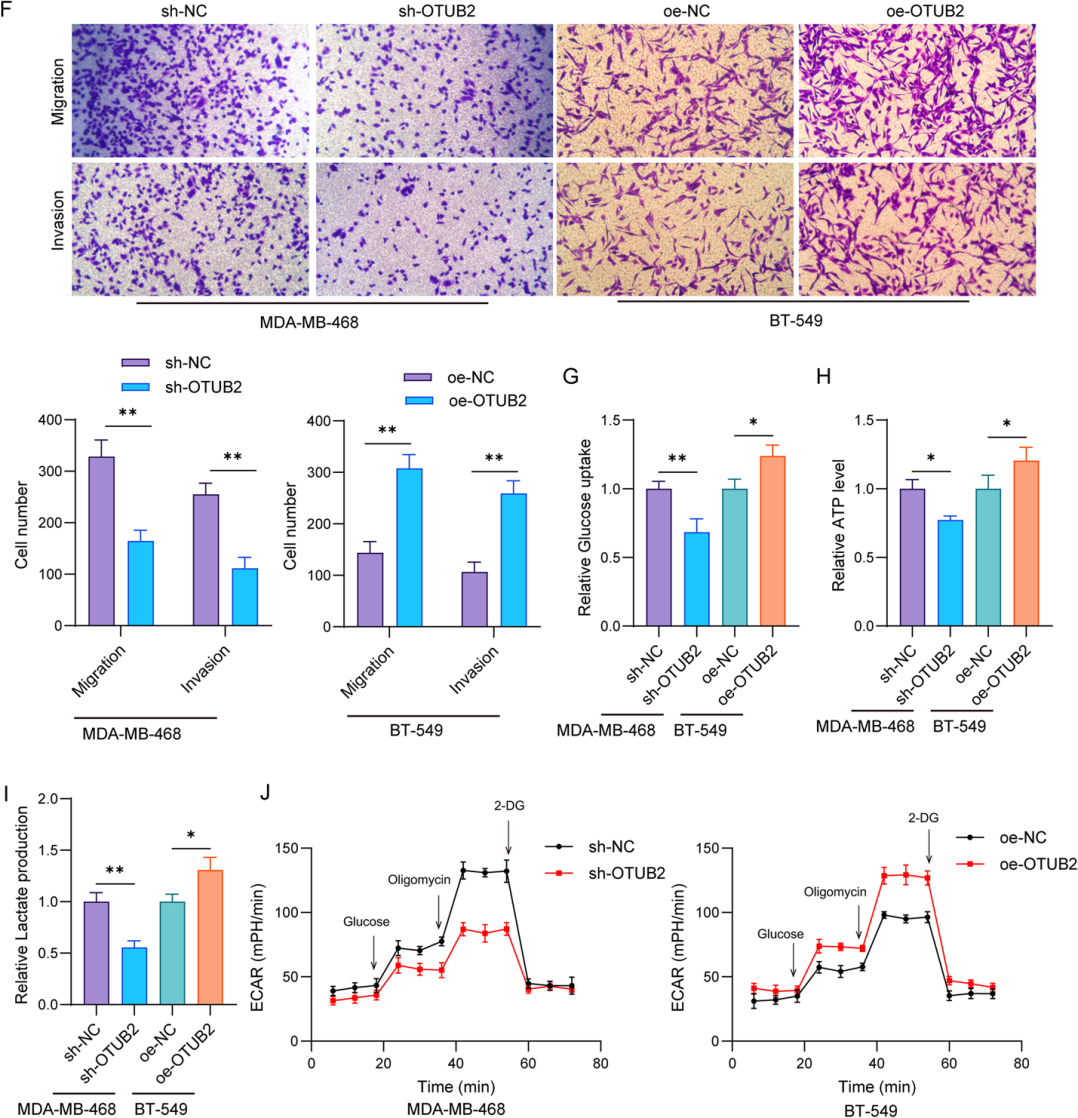
OTUB2 facilitates the malignant activities of TNBC cells. **A** and **B** MDA-MB-468 cells were infected with sh-OTUB2#1/2/3 or sh-NC, and BT-549 cells were infected with oe-OTUB2 or oe-NC, respectively. qPCR and western blot were performed to confirm the infection efficiency. MDA-MB-468 cells were infected with sh-OTUB2#2 or sh-NC, and BT-549 cells were infected with oe-OTUB2 or oe-NC, respectively. *p < 0.05; **p < 0.01; ***p < 0.001. **C** CCK-8, **D** colony-forming, **E** EdU, and **F** Transwell assays were employed to assess cell proliferation, migration, and invasion. **p < 0.01; ***p < 0.001. **G** Glucose uptake ratio was measured by the colorimetric assay kit. *p < 0.05; **p < 0.01. **H** ATP level and **I**)lactate production was measured by corresponding kits. *p < 0.05; **p < 0.01. **J** The ECAR of cells was analyzed using ECAR assay. *n* = 3 per group; Results are expressed as mean ± SD. Statistical analyses were analyzed using one-way ANOVA followed by Tukey’s multiple-comparisons test in A, B, and F-I. A Student’s t-test was used to analyze differences between two groups in C-E, and J

Three shRNAs and overexpression vectors were employed to knockdown or overexpress OTUB2 in MDA-MB-468 cells or BT-549 cells, respectively. After infection with sh-OTUB2#1/2/3, the expressions of OTUB2 mRNA and protein were significantly downregulated in MDA-MB-468 cells. Among these, sh-OTUB2#2 demonstrated the most pronounced infection efficiency and was thus selected for subsequent experimental procedures. In addition, OTUB2 mRNA and protein expressions were markedly increased in BT-549 cells after intervention with oe-OTUB2, suggesting the effective overexpression (Fig. 2A and B). Cell proliferation and colony formation were declined in sh-OTUB2-infected MDA-MB-468 cells compared to cells expressing sh-NC, as demonstrated by CCK-8, colony-forming, and EdU assays. In contrast, oe-OTUB2 infection in BT-549 cells led to an increase in cell proliferation and colony formation (Fig. 2C–E & Supplementary Fig. 1A–C). Consistently, cell migration and invasion were reduced in cells with OTUB2 silencing compared to cells with control vectors. The OTUB2 overexpression promoted cell migration and invasion (Fig. 2F & Supplementary Fig. 1D). Moreover, the knockdown of OTUB2 decreased glucose uptake, ATP level, and lactate production in MDA-MB-468 cells, whereas the OTUB2 upregulation had an adverse effect (Fig. 2G and I). Also, ECAR assay revealed that the ECAR was attenuated in MDA-MB-468 cells after downregulation of OTUB2, while the upregulated OTUB2 in BT-549 cells enhanced the ECAR (Fig. 2J). Together, these data demonstrated that OTUB2 promoted cell proliferation, migration, invasion, and glycolysis in TNBC cells. 

### OTUB2 stabilizes EIF4A3 by deubiquitination

**Fig. 3:**
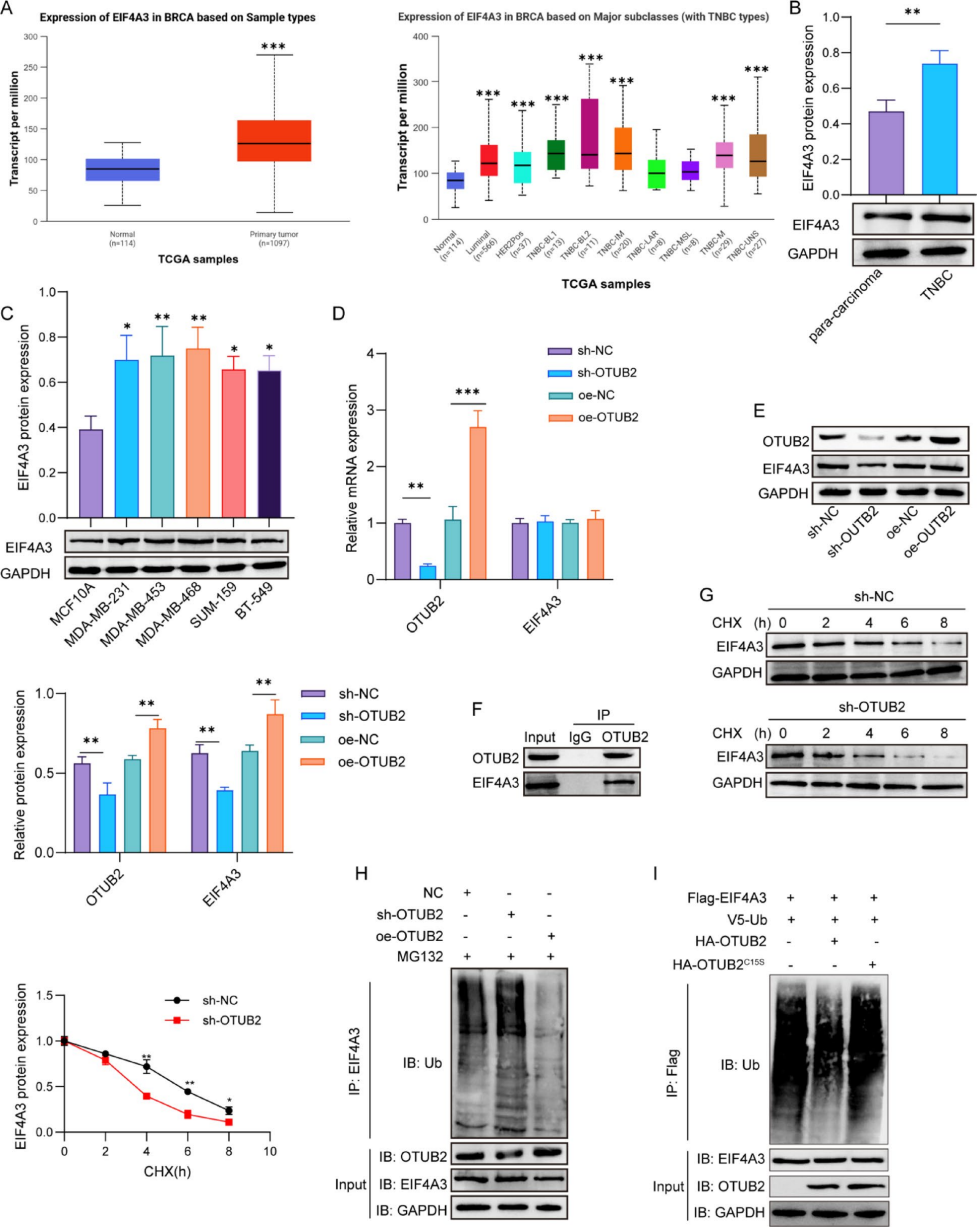
OTUB2 stabilizes EIF4A3 by deubiquitination. **A** TCGA database was used to analyze EIF4A3 expression in breast cancer tissues and TNBC tissues. ***p < 0.001. **B** Western blot detected the expression of EIF4A3 in TNBC tissues and para-carcinoma tissues (*n* = 30 samples per group). **p < 0.01. **C** Western blot detected the EIF4A3 protein in MCF10A, MDA-MB-231, MDA-MB-453, MDA-MB-468, SUM-159, and BT-549 cells. *p < 0.05; **p < 0.01. **D** and **E** MDA-MB-468 cells were infected with sh-OTUB2, sh-NC, oe-OTUB2, and oe-NC, respectively. Relative mRNA and protein expressions of OTUB2 and EIF4A3 were detected. **p < 0.01; ***p < 0.001. **F** Co-IP assay for measurement of interaction between OTUB2 and EIF4A3. OTUB2 and EIF4A3 protein expressions were evaluated in anti-OTUB2 complex or anti-IgG by western blot. **G** MDA-MB-468 cells infection with sh-OTUB2 or sh-NC were treated with CHX for 0, 2, 4, 6, 8 h, respectively. EIF4A3 protein was measured by western blot. *p < 0.05; **p < 0.01. **H** MDA-MB-468 cells infection with sh-OTUB2 or oe-OTUB2 were treated with MG132 for 6 h. Ubiquitination assay was used to test the effect of OTUB2 on EIF4A3 ubiquitination. **I** In vitro ubiquitination assay. Purified V5-ubiquitin and Flag-EIF4A3 were incubated with either HA-OTUB2 or HA-OTUB2^C51S^. Then, Flag-U2AF2 proteins were further purified with anti-Flag antibody and immunoblotted with antibodies against V5 and Flag. *n* = 3 per group; Results are expressed as mean ± SD. Statistical analyses were analyzed using a Student’s t-test in A (left) and B, while one-way ANOVA followed by Tukey’s multiple-comparisons test were used to analyze differences between groups in A(right), C-E, and G

The TCGA analysis data showed that EIF4A3 expression was increased in breast cancer tissues and TNBC tissues (Fig. 3A). Accordingly, western blot assay confirmed the upregulation of EIF4A3 protein in TNBC tissues (Fig. 3B). Compared with MCF10A cells, EIF4A3 protein expression was increased in TNBC cell lines, with the highest expression in MDA-MB-468 cells and the lowest expression in BT-549 cells (Fig. 3C). Further data showed that OTUB2 silencing remarkably reduced OTUB2 mRNA expression, and OTUB2 overexpressing enhanced OTUB2 mRNA expression in MDA-MB-468 cells. However, either OTUB2 silencing or overexpressing had no significant effect on EIF4A3 mRNA expression (Fig. 3D). Differently, OTUB2 silencing reduced OTUB2 and EIF4A3 protein levels, and OTUB2 overexpression enhanced OTUB2 and EIF4A3 protein levels (Fig. 3E). As revealed by the Co-IP assay, the EIF4A3 protein level was more in anti-OTUB2 immunoprecipitates than the anti-IgG or input (Fig. 3F), confirming an interaction between OTUB2 and EIF4A3. To further determine whether OTUB2 regulated the EIF4A3 protein stability, a CHX assay was conducted. The results indicated that the degradation of EIF4A3 protein was accelerated after the knockdown of OTUB2 in MDA-MB-468 cells (Fig. 3G). Since the protein stability was related to the ubiquitin-proteasome system, we assessed the effect of OTUB2 on EIF4A3 ubiquitination. As revealed, OTUB2 knockdown enhanced the ubiquitination level of EIF4A3, while its overexpression inhibited EIF4A3 ubiquitination (Fig. 3H). To determine whether OTUB2 directly deubiquitinates EIF4A3, we utilized a catalytic mutant, HA-OTUB2^C51S^, which is deficient in deubiquitinase activity. In an in vitro assay, ubiquitinated EIF4A3 purified from 293T cells was incubated with purified wild-type OTUB2 or the OTUB2^C51S^ mutant. The results showed that OTUB2, but not the catalytically inactive OTUB2^C51S^, effectively reduced the levels of ubiquitinated EIF4A3 (Fig. 3I). Therefore, OTUB2 enhanced the stability of EIF4A3 protein via its deubiquitination in TNBC cells.

### OTUB2 promotes malignant progression of TNBC cells through regulation of EIF4A3

**Fig. 4:**
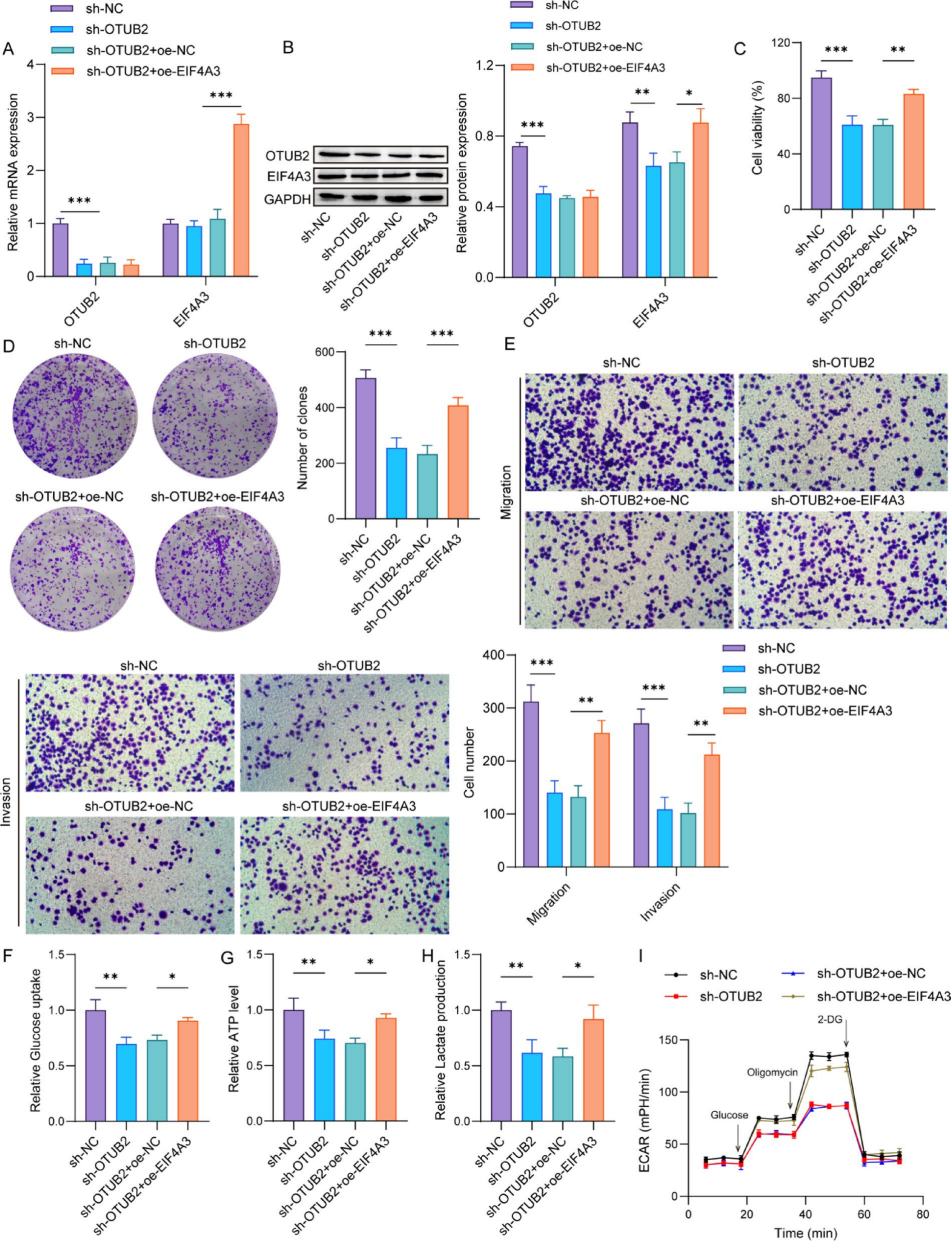
OTUB2 regulates EIF4A3 to promote malignant progression of TNBC cells. MDA-MB-468 cells were infected with sh-OTUB2 or co-infected with sh-OTUB2 and oe-EIF4A3. **A** and **B** qPCR and western blot were used to detect the mRNA and protein expressions of OTUB2 and EIF4A3. *p < 0.05; **p < 0.01; ***p < 0.001. **C** CCK-8, **D** colony-forming, and **E** Transwell assays were performed to evaluate cell proliferation, migration and invasion. **p < 0.01; ***p < 0.001. **F** Glucose uptake, **G** ATP level, and **H** lactate production in MDA-MB-468 cells were measured by corresponding kits. *p < 0.05; **p < 0.01. **I** The ECAR of cells was analyzed using ECAR assay. *n* = 3 per group; Results are expressed as mean ± SD. Statistical analyses were analyzed using one-way ANOVA followed by Tukey’s multiple-comparisons test

To examine whether OTUB2 regulates malignant progression of TNBC cells via EIF4A3, MDA-MB-468 cells were infected with sh-OTUB2 or co-infected with sh-OTUB2 and oe-EIF4A3. OTUB2 knockdown reduced OTUB2 mRNA expression, while did not affect EIF4A3 mRNA expression. The OTUB2 knockdown plus EIF4A3 overexpression had no significant effect on OTUB2 mRNA, while upregulated EIF4A3 mRNA expression (Fig. 4A). Differently, OTUB2 knockdown reduced both OTUB2 and EIF4A3 protein levels, and OTUB2 knockdown plus EIF4A3 overexpression led to an increase in EIF4A3 protein without any effect on the OTUB2 protein (Fig. 4B). The proliferation, colony formation, migration and invasion were decreased in OTUB2 silencing cells, whereas these effects were negated by EIF4A3 overexpression (Fig. 4C–E). Additionally, the knockdown of OTUB2 decreased glucose uptake, ATP level, and lactate production in MDA-MB-468 cells, whereas the EIF4A3 overexpression reversed these changes (Fig. 4F–H). Finally, an ECAR assay demonstrated that ECAR was attenuated in OTUB2 silencing cells, while the EIF4A3 overexpression abrogated this change (Fig. 4I). These data suggested that OTUB2 promoted the malignant progression of TNBC cells via upregulating EIF4A3 protein.

### EIF4A3 stabilizes TPI1 mRNA expression

**Fig. 5:**
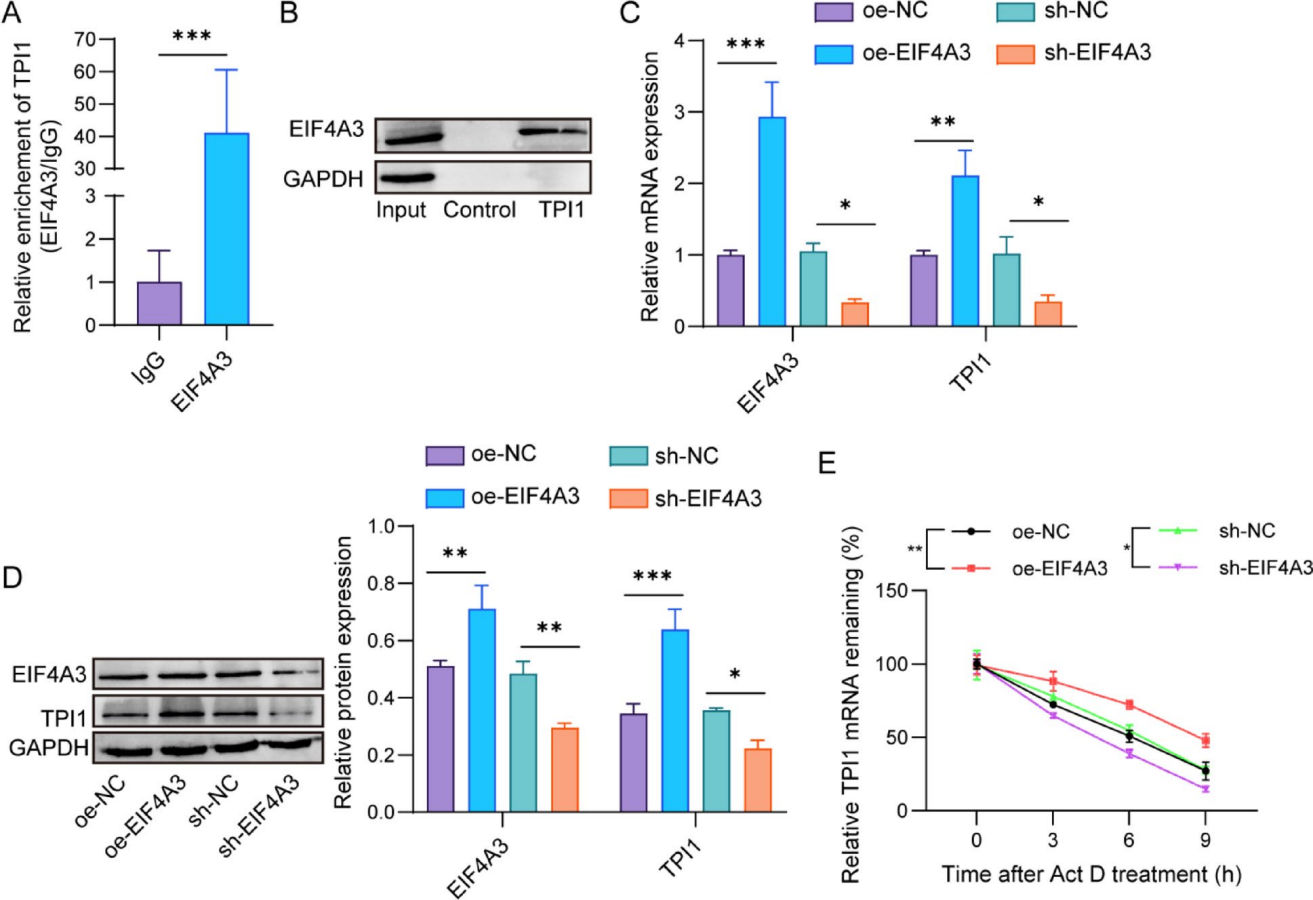
EIF4A3 stabilized TPI1 mRNA expression. **A** RIP assay was carried out to determine the interaction of EIF4A3 and TPI1. ***p < 0.001. **B** RNA pull down assay for the identification of EIF4A3 interacting with TPI1. **C** and **D** MDA-MB-468 cells were infected with oe-EIF4A3, oe-NC, sh-EIF4A3, and sh-NC, respectively. qPCR and western blot analysis detected EIF4A3 and TPI1 mRNA and protein expressions. *p < 0.05; **p < 0.01; ***p < 0.001. **E** MDA-MB-468 cells were infected with oe-EIF4A3, oe-NC, sh-EIF4A3, and sh-NC, following by actinomycin D treatment for 0, 3, 6, and 9 h. Relative TPI1 mRNA level was measured. *p < 0.05, **p < 0.01; *n* = 3 per group; Results are expressed as mean ± SD. Statistical analyses were analyzed using a Student’s t-test in A, while one-way ANOVA followed by Tukey’s multiple-comparisons test were used to analyze differences between groups in C-E

To further confirm the protein interaction between EIF4A3 and TPI1, RIP assay was conducted. As shown in Fig. 5A and Supplementary Fig. 2A, the enrichment of TPI1 was more in anti-EIF4A3 immunoprecipitates than anti-IgG in 293T cells and MDA-MB-468 cells. Then, the RNA pull down assay also verified the interaction between EIF4A3 and TPI1, as evidenced by the increased EIF4A3 protein in TPI1 complex (Fig. 5B & Supplementary Fig. 2B). Meanwhile, qPCR and western blot analysis revealed that overexpression of EIF4A3 elevated EIF4A3 and TPI1 mRNA and protein levels, and the knockdown of EIF4A3 emerged an adverse effect (Fig. 5C&D). Actinomycin D was added to cells to inhibit the mRNA synthesis. It was found that EIF4A3 overexpressing enhanced the TPI1 mRNA stability, but the EIF4A3 knockdown declined the TPI1 mRNA stability (Fig. 5E). Above data revealed that EIF4A3 stabilized TPI1 mRNA expression. EIF4A3 promoted malignant progression of TNBC cells through TPI1-mediated glycolysis.

**Fig. 6:**
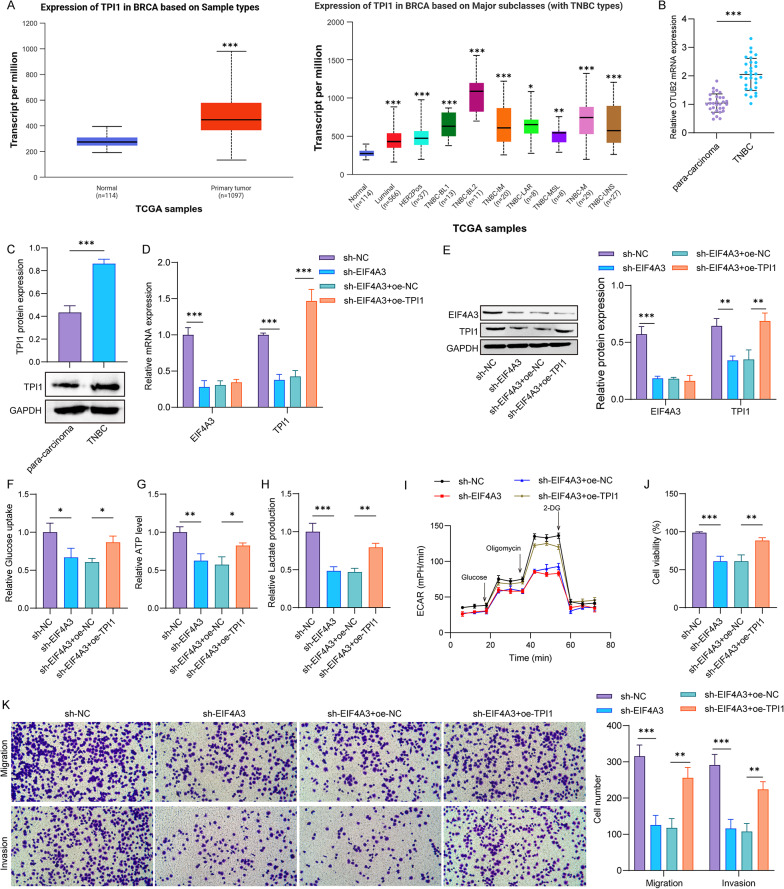
EIF4A3 promotes malignant progression of TNBC cells through TPI1-mediated glycolysis. **A** TPI1 expression analysis in breast cancer tissues and TNBC tissues using TCGA database. *p < 0.05; **p < 0.01; ***p < 0.001. **B** and **C** qPCR and western blot analysis of TPI1 mRNA and protein in TNBC tissues and para-carcinoma tissues (*n* = 30 samples per group). ***p < 0.001. **D** and **E** MDA-MB-468 cells were infected with sh-EIF4A3 or co-infected with sh-EIF4A3 and oe-TPI1. Relative mRNA and protein expressions of EIF4A3 and TPI1 were detected by qPCR and western blot. *p < 0.05; **p < 0.01; ***p < 0.001. **F** Glucose uptake, **G** ATP level, and **H** lactate production in MDA-MB-468 cells were measured by corresponding kits. *p < 0.05; **p < 0.01; ***p < 0.001. **I** The ECAR of cells was analyzed using ECAR assay. **J** CCK-8 and **K** Transwell assays were performed to evaluate cell proliferation, migration and invasion. **p < 0.01; ***p < 0.001. *n* = 3 per group; Results are expressed as mean ± SD. Statistical analyses were analyzed using a Student’s t-test in A (left), B, and C, while one-way ANOVA followed by Tukey’s multiple-comparisons test were used to analyze differences between groups in A (right), and D-K

The TCGA analysis data explained that TPI1 was upregulated in breast cancer tissues and TNBC tissues (Fig. 6A). qPCR and western blot assays revealed the increased TPI1 mRNA and protein expressions in TNBC tissues (Fig. 6B&C). Then, the EIF4A3 silencing reduced EIF4A3 and TPI1 mRNA and protein expressions, and the TPI1 overexpression enhanced TPI1 mRNA and protein, but had no significant influence on EIF4A3 mRNA and protein expressions (Fig. 6D&E). Furthermore, EIF4A3 deletion decreased glucose uptake, ATP level, and lactate production in MDA-MB-468 cells, whereas the TPI1 overexpression reversed these effects (Fig. 6F-H). Similarly, ECAR was attenuated in EIF4A3 silencing cells, which was then enhanced in cells with TPI1 overexpression (Fig. 6I). Additionally, cell proliferation, migration, and invasion were reduced after EIF4A3 knockdown, while the TPI1 overexpression restored these changes (Fig. 6J&K). In sum, these data suggested that EIF4A3 promoted malignant progression of TNBC through TPI1-mediated glycolysis.

### OTUB2 promotes malignant progression of TNBC cells via EIF4A3/TPI1-mediated glycolysis

**Fig. 7:**
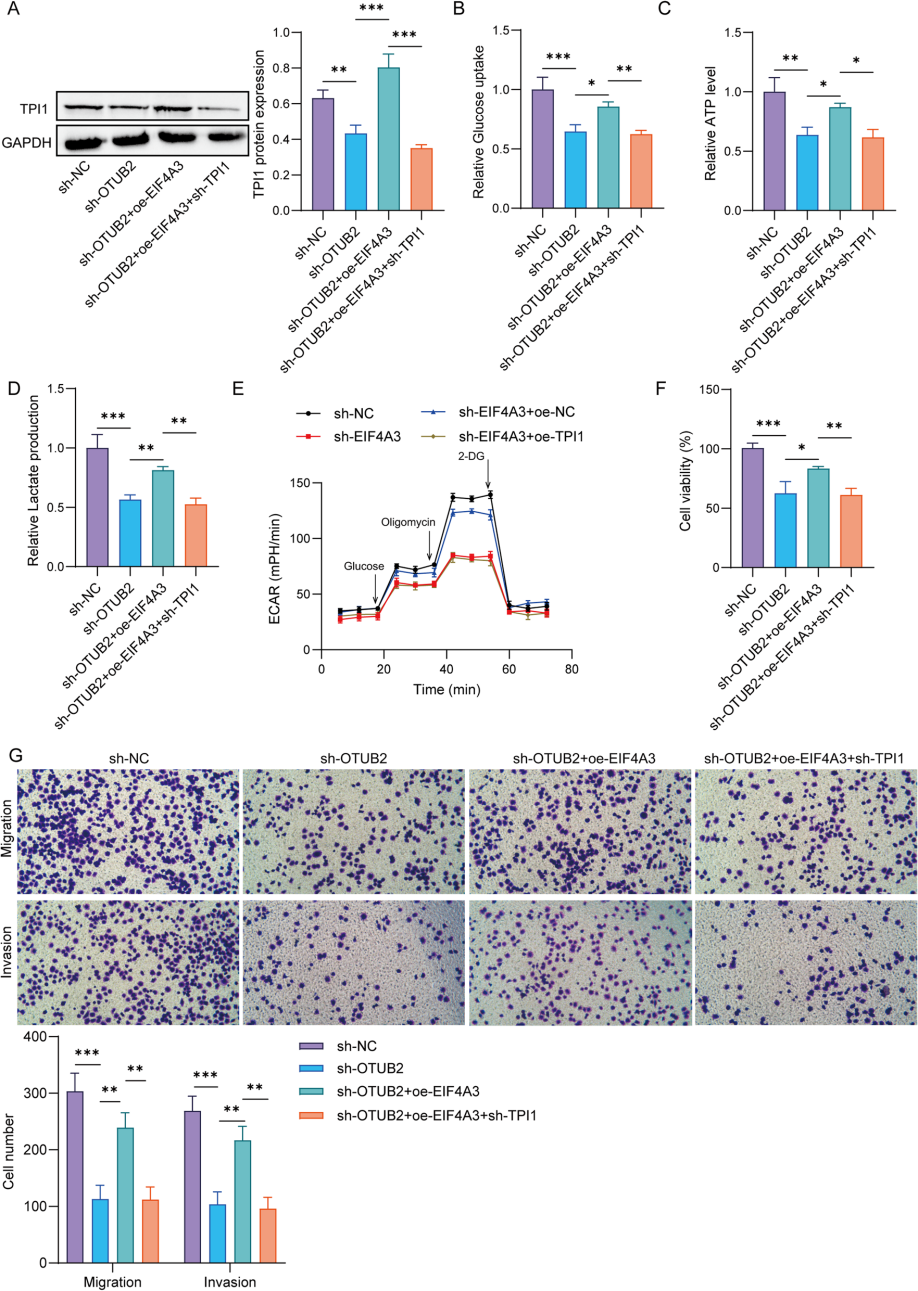
OTUB2 promotes malignant progression of TNBC cells through EIF4A3/TPI1 mediated glycolysis. The infected or co-infected MDA-MB-468 cells were grouped as follows: sh-NC, sh-OTUB2, sh-OTUB2 + oe-EIF4A3, and sh-OTUB2 + oe-EIF4A3 + sh-TPI1. **A** Western blot detection of TPI1 protein. **p < 0.01; ***p < 0.001. **B** Glucose uptake, **C** ATP level, and **D** lactate production was measured by corresponding kits. *p < 0.05; **p < 0.01; ***p < 0.001. **E** The ECAR of MDA-MB-468 cells was analyzed using ECAR assay. **F** CCK-8 and **G** Transwell assays were used to evaluate cell proliferation, migration, and invasion. *p < 0.05; **p < 0.01; ***p < 0.001. *n* = 3 per group; Results are expressed as mean ± SD. Statistical analyses were analyzed using an one-way ANOVA followed by Tukey’s multiple-comparisons test

Given the above results, we further explored whether OTUB2 affects malignant progression of TNBC cells through the EIF4A3/TPI1-mediated glycolysis. As shown in Fig. 7A, OTUB2 knockdown led to a decrease in TPI1 protein expression, and EIF4A3 overexpression elevated the TPI1 protein, while the further TPI1 silencing declined TPI1 protein expression. Further data showed that OTUB2 knockdown restrained the glucose uptake, ATP level, and lactate production in MDA-MB-468 cells, while the EIF4A3 overexpression restored these changes. However, the elevated glucose uptake, ATP level, and lactate production induced by EIF4A3 overexpression were inhibited by TPI1 knockdown (Fig. 7B-D). Meanwhile, inhibition of OTUB2 attenuated the ECAR of MDA-MB-468 cells, and the EIF4A3 overexpression led to an increase in ECAR, which was then reversed by TPI1 deletion (Fig. 7E). In addition, OTUB2 silencing lessened cell proliferation, migration and invasion, which were then negated by overexpression of EIF4A3. Whereas, these elevated cell malignant activities caused by EIF4A3 overexpression were suppressed by TPI1 knockdown (Fig. 7F&G). In BT-549 cells, overexpression of OTUB2 facilitates cell viability and clone formation, as well as enhanced lactate production and ECAR, which was then declined by EIF4A3 silencing or TPI1 knockdown (Supplementary Fig. 3A-D). Additionally, in MDA-MB-468 cells, overexpression of TPI1 reversed the effect of OTUB2 knockdown on cell viability, clone formation, lactate production and ECAR (Supplementary Fig. 4A-D). Thus, these data indicated that OTUB2 accelerated TNBC cell malignant activities through EIF4A3/TPI1-mediated glycolysis.

### OTUB2 facilitates TNBC malignant progression via EIF4A3/TPI1 in vivo

**Fig. 8:**
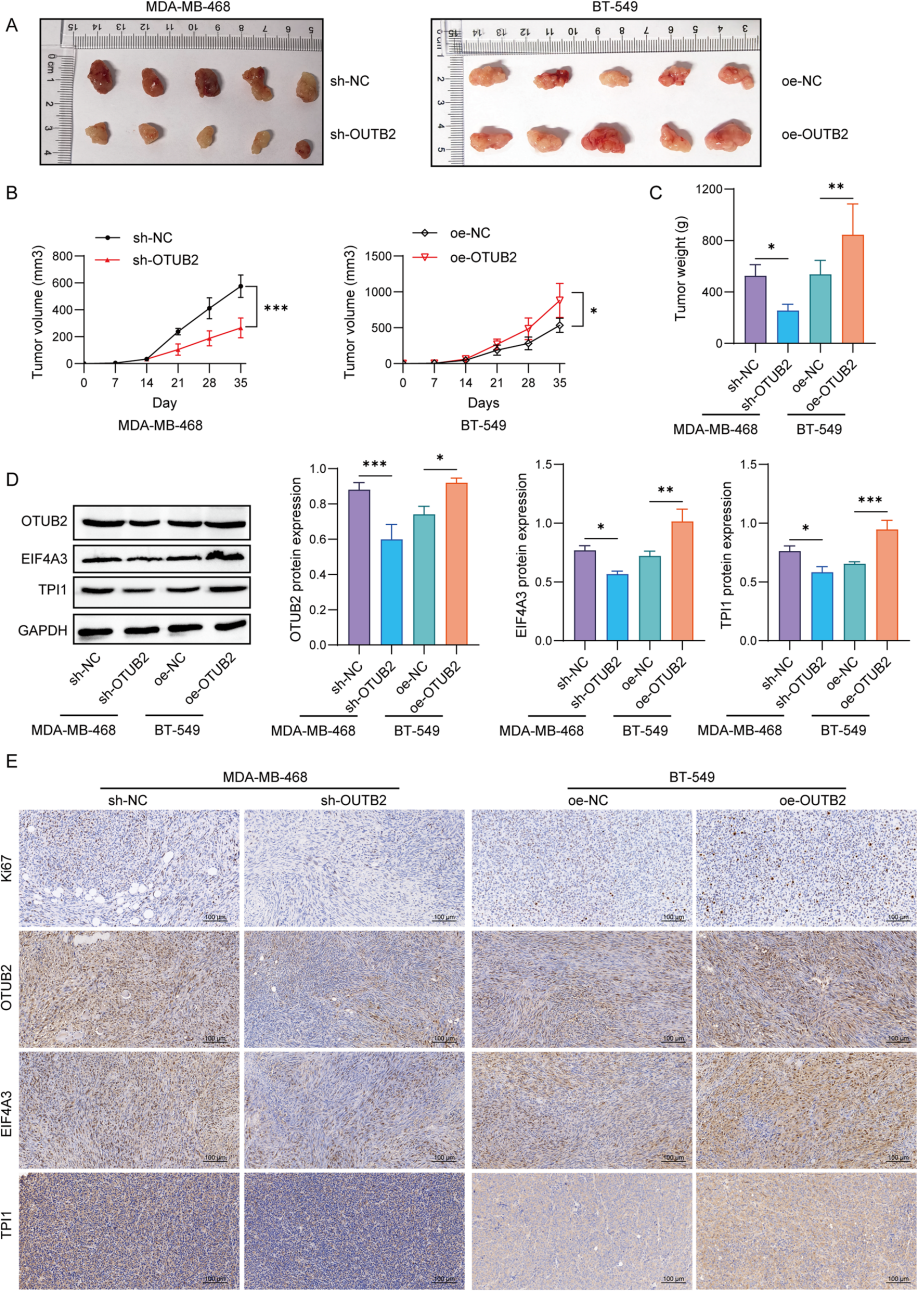
OTUB2 facilitates TNBC malignant progression via EIF4A3/TPI1 in vivo. MDA-MB-468 infected with sh-OTUB2 or sh-NC, and BT-549 cells infected with oe-OTUB2 or oe-NC were subcutaneously injected into the right thoracic mammary fat pads of nude mice, respectively (*n* = 5 mice per group). **A** Tumor images. **B** Tumor volume was measured every 7 days. *p < 0.05;***p < 0.001. **C** Tumor weight was recorded after 35 days. *p < 0.05; **p < 0.01. **D** The protein expressions of OTUB2, EIF4A3, and TPI1 were detected. **E** The Ki-67, OTUB2, EIF4A3, and TPI1 proteins were evaluated using immumohistochemical assay. *p < 0.05; **p < 0.01; ***p < 0.001. Results are expressed as mean ± SD. Statistical analyses were analyzed using a Student’s t-test in B, while one-way ANOVA followed by Tukey’s multiple-comparisons test were used to analyze differences between groups in C and D

Based on the aforementioned results, we established the mouse xenograft model to validate the role of OTUB2 in TNBC. Our findings revealed that, compared to the sh-NC group, the sh-OTUB2 group exhibited a significant reduction in both tumor volume and weight. Conversely, the oe-OTUB2 group demonstrated a notable increase in both tumor volume and weight when compared to the oe-NC group (Fig. 8A-C). Also, OTUB2, EIF4A3, and TPI1 protein expressions were reduced in the tumor tissues of the sh-OTUB2 group in comparison with the sh-NC group, but these protein expressions showed a reverse trend in oe-OTUB2 group (Fig. 8D). Consistently, the immumohistochemical staining data revealed that the Ki-67, OTUB2, EIF4A3, and TPI1 proteins were decreased in tumor tissues of the sh-OTUB2 group relative to the sh-NC group, and the oe-OTUB2 group showed an adverse trend (Fig. 8E). These results suggested that OTUB2 promoted TNBC malignant progression via glycolysis mediated by EIF4A3/TPI1 axis.

## Discussion

Breast cancer has the highest occurrence among all cancer types in women, with TNBC representing a particularly malignant and aggressive subtype. Thus, exploring the pathogenesis of TNBC is of great significance. In our study, by analyzing TCGA database, OTUB2, EIF4A3, and TPI1 were highly expressed in TNBC tissues, and this finding was further validated in TNBC patients and cell lines. Specifically, OTUB2 deubiquitinated and stabilized EIF4A3 protein, which in turn stabilized TPI1 mRNA. EIF4A3 overexpression exacerbated TNBC malignant activities and glycolysis, while this effect was then mitigated by TPI1 knockdown. Moreover, OTUB2 silencing inhibited tumor growth in a xenograft model. Our study, for the first time, demonstrates the role of OTUB2/EIF4A3/TPI1 axis in promoting TNBC progression and offers a novel approach for targeted glycolysis therapy.

OTUB2, a well-established deubiquitinating enzyme, has been extensively implicated in tumor progression. For instance, Ouyang et al. demonstrated that OTUB2 regulated KRT80 stability through deubiquitination, thereby promoting tumor proliferation in gastric cancer [21]. Chang et al. revealed that OTUB2 promoted the deubiquitination and phosphorylation of STAT1, leading to CALML3 activation and exerting tumor-suppressive roles in oral and esophageal squamous cell carcinomas [22]. Strikingly, a recent study found increased OTUB2 expression in TNBC xenograft models and cell lines [23], suggesting its involvement in TNBC progression. EIF4A3 is a pivotal component of the exon junction complex, functioning as a crucial regulator in various post-transcriptional processes, including mRNA splicing, transport, and translation [24]. Studies have shown the carcinogenic effects of EIF4A3 in various cancers, including TNBC [25, 26]. Our analysis of the TCGA database, along with experimental data, revealed upregulated OTUB2 and EIF4A3 expression in TNBC tissues and cell lines. Functionally, we found that knockdown of OTUB2 significantly suppressed cell proliferation, colony formation, migration, and invasion, while OTUB2 overexpression exhibited opposite effects. These findings suggest that OTUB2 plays an oncogenic role in TNBC progression and may serve as a therapeutic target for TNBC patients. Energy metabolism in cancer has been extensively studied, with glycolysis emerging as an attractive therapeutic target due to the significant increase in glucose uptake in many tumors [27]. Enhanced glycolysis in cancer cells leads to lactate accumulation, which correlates with malignant progression and poor prognosis in various human cancers, including TNBC [28]. Our study found that OTUB2 knockdown decreased glucose uptake, ATP levels, lactate production, and ECAR, while OTUB2 overexpression showed a reverse trend. Mechanistically, we demonstrated that OTUB2 stabilized the EIF4A3 protein via deubiquitination. As a crucial regulatory factor of protein ubiquitination, OTUB2 exhibits specificity in recognizing polyubiquitinated chains with distinct junction types. It removes the polyubiquitin chains from target genes via its active site, ultimately affecting the stability of downstream targets [29]. This study extends previous findings by identifying EIF4A3 as a novel substrate of OTUB2 in TNBC, indicating that OTUB2 may have broader oncogenic functions via stabilization of key proteins. Our data suggest that OTUB2 promotes glycolysis and TNBC progression, potentially through EIF4A3 deubiquitination and stabilization.

Recent studies have highlighted the significant role of EIF4A3 in cancer progression, making it a potential therapeutic target. Dysregulation of EIF4A3 has been linked to various cancers, including lung cancer [30], colorectal cancer [31], and endometrial cancer [32], et al. Furthermore, EIF4A3 was found remarkably upregulated in breast cancer, with this elevation associated with a poor prognosis [33]. In our current study, we observed that EIF4A3 overexpression potentiated the proliferation, colony formation, migration, and invasion of OTUB2-silenced cells. Additionally, EIF4A3 overexpression reversed the inhibitory effects of OTUB2 knockdown on cellular glucose uptake, ATP levels, lactate production, and ECAR. These findings further support our hypothesis that OTUB2 promotes glycolysis and malignant progression in TNBC cells through the stabilization and upregulation of the EIF4A3 protein. Moreover, EIF4A3 was one of the RBPs interacting with transcripts to form a complex, thereby affecting the target mRNA stability and expression. As proof, Xia et al. revealed that circMYH9 enhanced KPNA2 mRNA stability to accelerate hepatocellular carcinoma development by interacting with RBP EIF4A3 [34]. Ren et al. SNHG16 modulated RhoU expression by recruiting EIF4A3 to enhance the mRNA stability of RhoU [35]. These evidences suggest that OTUB2–EIF4A3 pathway may regulate target mRNA stability.

Energy metabolism in tumors has been extensively explored. Glycolysis represents a promising therapeutic target, given the pronounced increase in glucose uptake seen in many tumors compared to adjacent normal tissue [36]. TPI1, a pivotal glycolytic enzyme, is frequently upregulated in numerous cancers, including breast cancer. As previously described, TPI1 predicts poor prognosis in breast cancer, and it plays a significant role in promoting breast cancer metastasis and glycolysis [17]. TPI1 catalyzes the reversible interconversion of dihydroxyacetone phosphate (DHAP) and glyceraldehyde‑3‑phosphate (GAP). Under conditions of low glucose availability, DHAP is converted to GAP, thereby enhancing glycolytic flux and promoting ATP production [37]. A recent study confirmed that downregulation of TPI1 inhibited glycolysis and suppressed the progression of TNBC [38]. However, the regulatory mechanism of TPI1 in TNBC remains poorly understood. EIF4A3 has been implicated in RNA interactions and identified as a potential diagnostic marker or therapeutic target for TNBC. For instance, EIF4A3 could combine with the upstream and downstream region of circSEPT9 pre-mRNA and modulate its expression in TNBC cells [39]. EIF4A3 was found to possess a binding site on the upstream region of circPRKCI transcript, and this interaction could induce circPRKCI expression, thereby facilitating the TNBC progression [40]. Our data indicated that TPI1 was highly expressed in TNBC tissues and cell lines, suggesting that TPI1 promotes tumors through its metabolic functions. In addition, TPI1 expression was positively regulated by EIF4A3 via interaction. Further results confirmed that EIF4A3 influenced the mRNA stability of TPI1. EIF4A3 overexpression counters the impact of OTUB2 silencing on TNBC malignant progression, an effect that is further abrogated by TPI1 deletion. In vivo, OTUB2 accelerated tumor growth in a mouse xenograft model. Our collectively data suggest that OTUB2 exacerbates TNBC malignant progression through EIF4A3/TPI1-mediated glycolysis. In the nearly recent, TPI1 undergoes ubiquitin-dependent proteasome degradation of downstream targets in breast cancer cells has been demonstrated [41]. The molecular mechanisms concerning TPI1-mediated ubiquitination of proteins in TNBC may be an interesting future research direction.

Nevertheless, it is important to acknowledge the limitations of our study. We have verified the binding of OTUB2 to EIF4A3, while the precise binding sites, including whether it interacts with the promoter region, remain to be elucidated in future studies. Furthermore, the mechanisms concerning the effects of OTUB2 on EIF4A3/TPI1 axis in vivo are still not fully understood and require further detailed investigation. Additionally, the sample size is small. Future research in a larger, well-annotated TNBC cohort should perform survival analyses and correlation analyses among OTUB2, EIF4A3, and TPI1; their expression should also be correlated with clinicopathologic features to determine the clinical and translational relevance.

In summary, our study reveals that OTUB2 plays an oncogenic role in TNBC. Specifically, we demonstrate that OTUB2 deubiquitinates and stabilizes EIF4A3, which in turn stabilizes TPI1 mRNA, leading to enhanced glycolysis and acceleration of TNBC malignant progression. Our findings suggest that targeting the OTUB2/EIF4A3/TPI1 axis may represent a novel therapeutic strategy for the treatment of TNBC.

## Supplementary Information

Below is the link to the electronic supplementary material.

Supplementary Material 1

Supplementary Material 2

Supplementary Material 3

Supplementary Material 4. OTUB2 knockdown attenuates the malignant activities of MDA-MB-468 cells. MDA-MB-468 cells were infected with sh-OTUB2#3 or sh-NC. (A) CCK-8, (B) colony-forming, (C) EdU, and (D) Transwell assays were employed to assess cell proliferation, migration, and invasion. *n* = 3 per group; Results are expressed as mean ± SD. Statistical analyses were analyzed using a Student’s t-test. *p < 0.05; ***p < 0.001

Supplementary Material 5. EIF4A3 interacts with TPI1 mRNA in MDA-MB-468 cells. (A-B) RIP and RNA pull down assays were carried out to determine the interaction between EIF4A3 and TPI1. ***p < 0.001. *n* = 3 per group; Results are expressed as mean ± SD. Statistical analyses were analyzed using a Student’s t-test

Supplementary Material 6. OTUB2 overexpression promotes the proliferation and glycolysis in BT-549 cells via regulation of EIF4A3/TPI1 pathway. BT-549 cells were subjected to the following treatments: infection with oe-OTUB2 or oe-NC; or co-infection with oe-OTUB2 and either sh-EIF4A3 or sh-TPI1. (A) CCK-8 and (B) colony-forming assays were employed to assess cell proliferation. (C) Lactate production was measured by commercial kits. (D) The ECAR of cells was analyzed using ECAR assay. *n* = 3 per group; Results are expressed as mean ± SD. Statistical analyses were analyzed using an one-way ANOVA followed by Tukey’s multiple-comparisons test. *p < 0.05; **p < 0.01; ***p < 0.001

Supplementary Material 7. OTUB2 silencing reduces the proliferation and glycolysis in MDA-MB-468 cells via regulation of TPI1. MDA-MB-468 cells were infected with sh-OTUB2 or sh-NC; or co-infected with sh-OTUB2 and oe-TPI1. (A-B) CCK-8 and colony-forming assays were employed to assess cell proliferation. (C-D) Lactate production and the ECAR of cells were detected. *n* = 3 per group; Results are expressed as mean ± SD. Statistical analyses were analyzed using an one-way ANOVA followed by Tukey’s multiple-comparisons test. **p < 0.01; ***p < 0.001

## Data Availability

The datasets used or analyzed during the current study are available from the corresponding author on reasonable request.

## Abbreviations

BCA: Bicinchoninic acid
CCK-8: Cell counting kit-8
CHX: Cycloheximide
Co-IP: Co-immunoprecipitation
DUBs: Deubiquitinases
ECAR: Extracellular acidification rate
ECL: Enhanced chemiluminiscent
EdU: 5-ethynyl-2′-deoxyuridine
EIF4A3: Eukaryotic initiation factor 4 A-III
ER: Estrogen receptor
FBS: Fetal bovine serum
HER2: Human epidermal growth factor receptor-2
OTUB2: Ovarian tumor domain ubiquitin aldehyde binding 2
PBS: Phosphate buffered saline
PR: Progesterone receptor
PVDF: Polyvinylidene fluoride
qPCR: Quantitative PCR
RBP: RNA binding protein
RIP: RNA immunoprecipitation
SDS-PAGE: Sodium dodecyl sulfate-polyacrylamide gel electrophoresis
TCGA: The Cancer Genome Atlas
TNBC: Triple-negative breast cancer
TPI1: Triosephosphate isomerase 1
