# Supplementary material for: Deubiquitination and stabilization of EIF4A3 by OTUB2 contributes to TPI1-mediated glycolysis and TNBC progression
Source: Breast Cancer Res. 2026 Mar 19;28:77. doi: 10.1186/s13058-026-02260-5 (PMC13123037; doi:10.1186/s13058-026-02260-5)
Supplement: Supplementary file 3 — Supplementary Material 3 [file 13058_2026_2260_MOESM3_ESM.docx]

1C. OTUB2







1C. GAPDH







1E. OTUB2







1E. GAPDH







2B. MDA-MB-468: OTUB2







2B. MDA-MB-468: GAPDH







2B. BT-549: OTUB2







2B. BT-549: GAPDH







3B. EIF4A3







3B. GAPDH







3C. EIF4A3







3C. GAPDH







3E. OTUB2







3E. EIF4A3







3E. GAPDH







3F. OTUB2







3F. EIF4A3







3G. sh-NC: EIF4A3







3G. sh-NC: GAPDH







3G. sh-OTUB2: EIF4A3







3G. sh-OTUB2: GAPDH







3H. Ub







3H. OTUB2







3H. EIF4A3







3H. GAPDH







3I. Ub







3I. EIF4A3







3I. OTUB2







3I. GAPDH







4B. OTUB2







4B. EIF4A3







4B. GAPDH







5B. EIF4A3







5B. GAPDH







5D. TPI1







5D. EIF4A3







5D. GAPDH







6C. TPI1







6C. GAPDH







6E. TPI1







6E. EIF4A3







6E. GAPDH







7A. TPI1







7A. GAPDH







8D. OTUB2







8D. TPI1







8D. EIF4A3







8D. GAPDH







S2-B. EIF4A3







S2-B. GAPDH
