# Supplementary figures and images for: Deubiquitination and stabilization of EIF4A3 by OTUB2 contributes to TPI1-mediated glycolysis and TNBC progression
Source: Breast Cancer Res. 2026 Mar 19;28:77. doi: 10.1186/s13058-026-02260-5 (PMC13123037; doi:10.1186/s13058-026-02260-5)

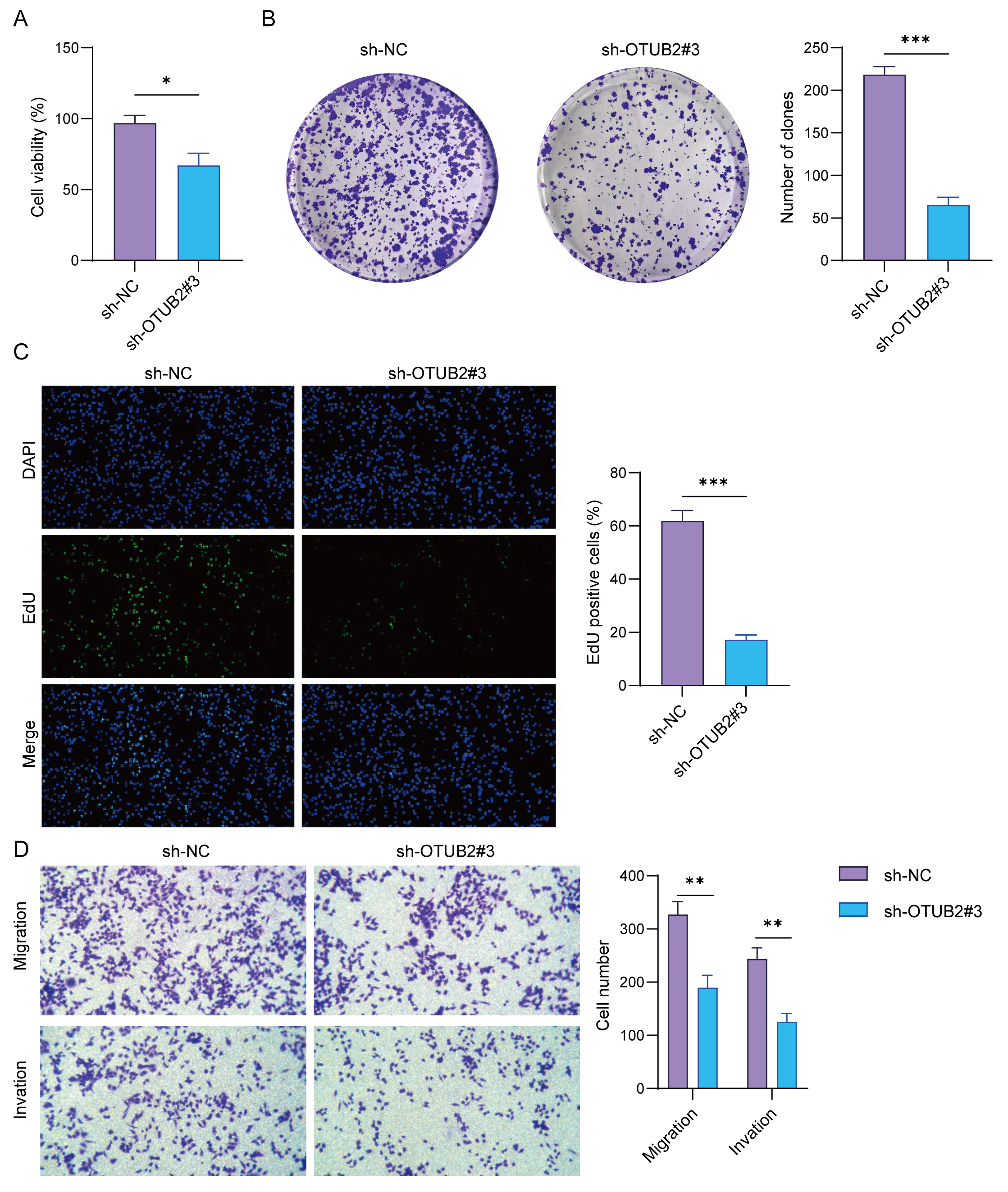

Supplement: Supplementary file 4 — Supplementary Material 4. OTUB2 knockdown attenuates the malignant activities of MDA-MB-468 cells. MDA-MB-468 cells were infected with sh-OTUB2#3 or sh-NC. (A) CCK-8, (B) colony-forming, (C) EdU, and (D) Transwell assays were employed to assess cell proliferation, migration, and invasion. n = 3 per group; Results are expressed as mean ± SD. Statistical analyses were analyzed using a Student’s t-test. *p < 0.05; ***p < 0.001 [file 13058_2026_2260_MOESM4_ESM.tif]

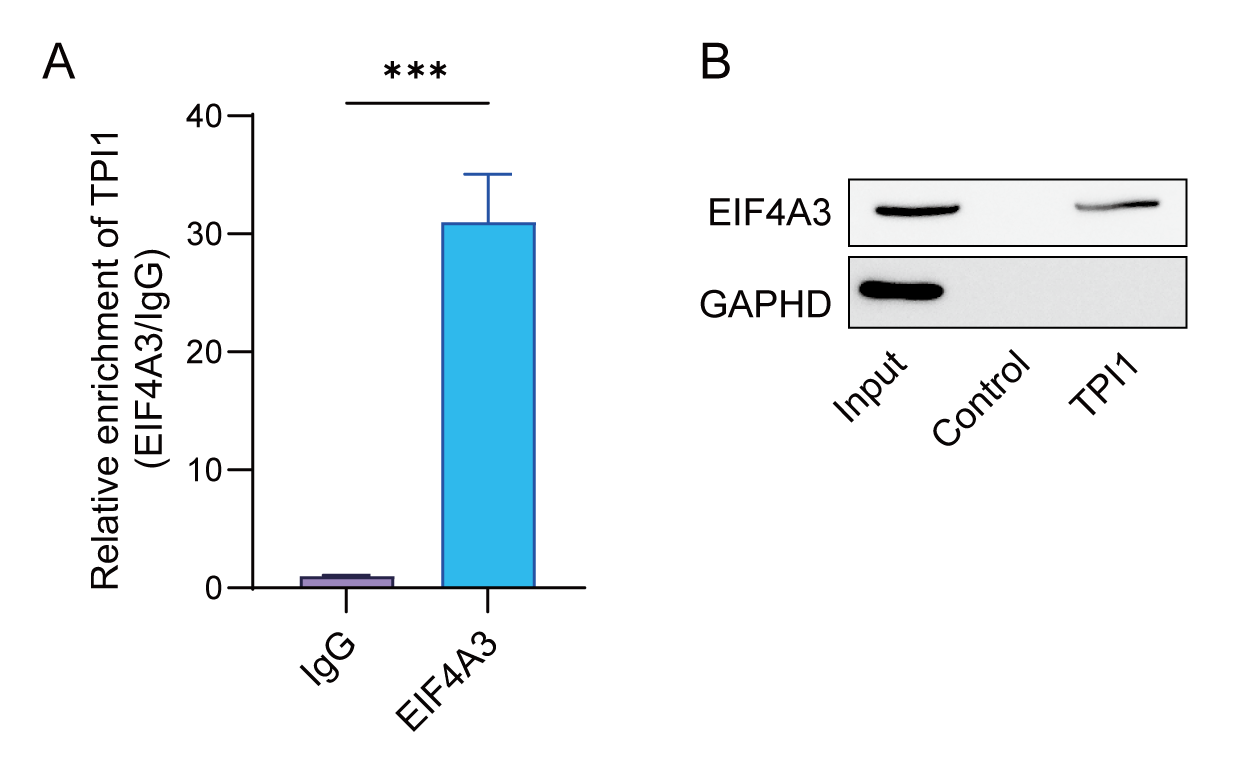

Supplement: Supplementary file 5 — Supplementary Material 5. EIF4A3 interacts with TPI1 mRNA in MDA-MB-468 cells. (A-B) RIP and RNA pull down assays were carried out to determine the interaction between EIF4A3 and TPI1. ***p < 0.001. n = 3 per group; Results are expressed as mean ± SD. Statistical analyses were analyzed using a Student’s t-test [file 13058_2026_2260_MOESM5_ESM.tif]

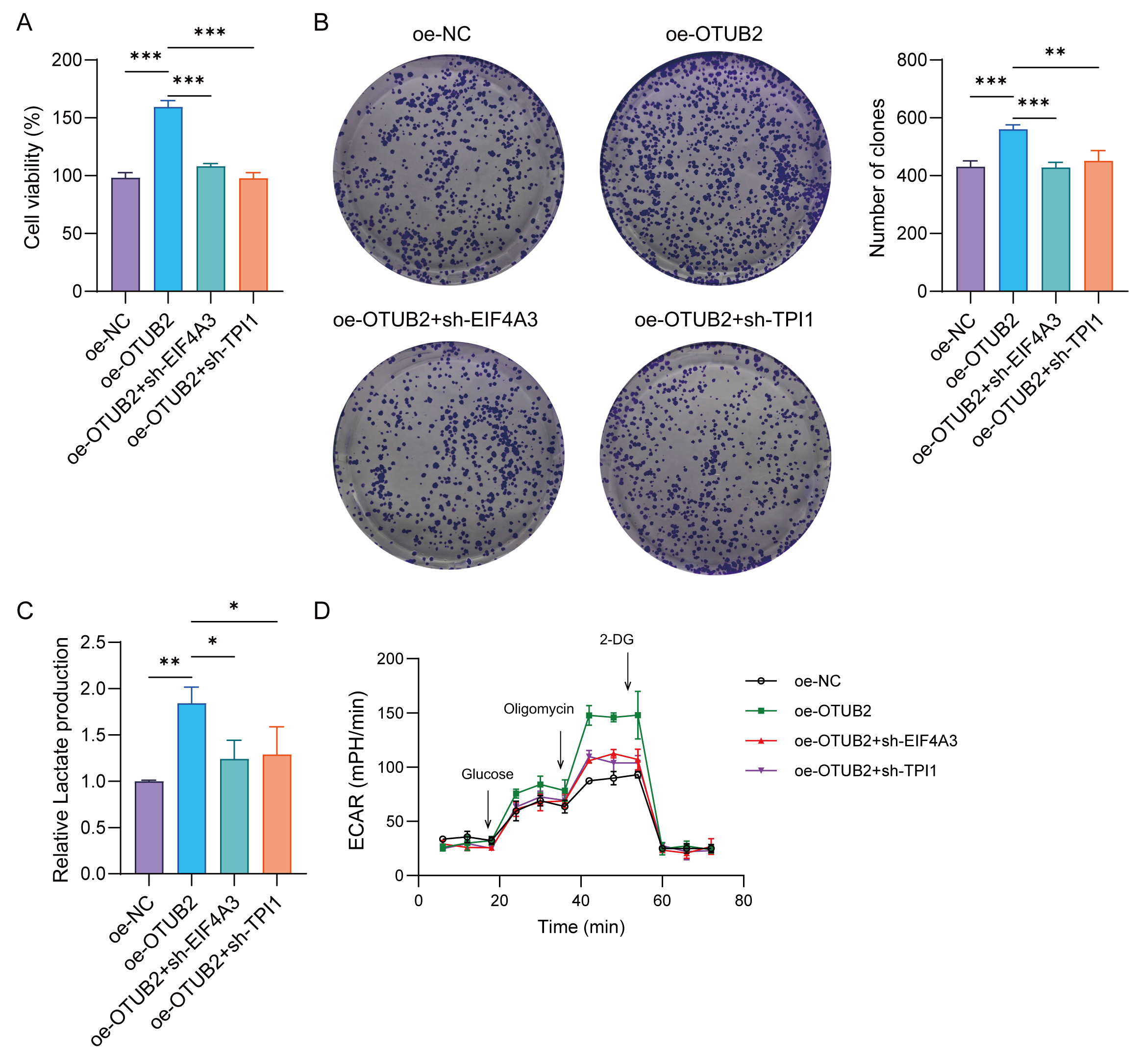

Supplement: Supplementary file 6 — Supplementary Material 6. OTUB2 overexpression promotes the proliferation and glycolysis in BT-549 cells via regulation of EIF4A3/TPI1 pathway. BT-549 cells were subjected to the following treatments: infection with oe-OTUB2 or oe-NC; or co-infection with oe-OTUB2 and either sh-EIF4A3 or sh-TPI1. (A) CCK-8 and (B) colony-forming assays were employed to assess cell proliferation. (C) Lactate production was measured by commercial kits. (D) The ECAR of cells was analyzed using ECAR assay. n = 3 per group; Results are expressed as mean ± SD. Statistical analyses were analyzed using an one-way ANOVA followed by Tukey’s multiple-comparisons test. *p < 0.05; **p < 0.01; ***p < 0.001 [file 13058_2026_2260_MOESM6_ESM.tif]

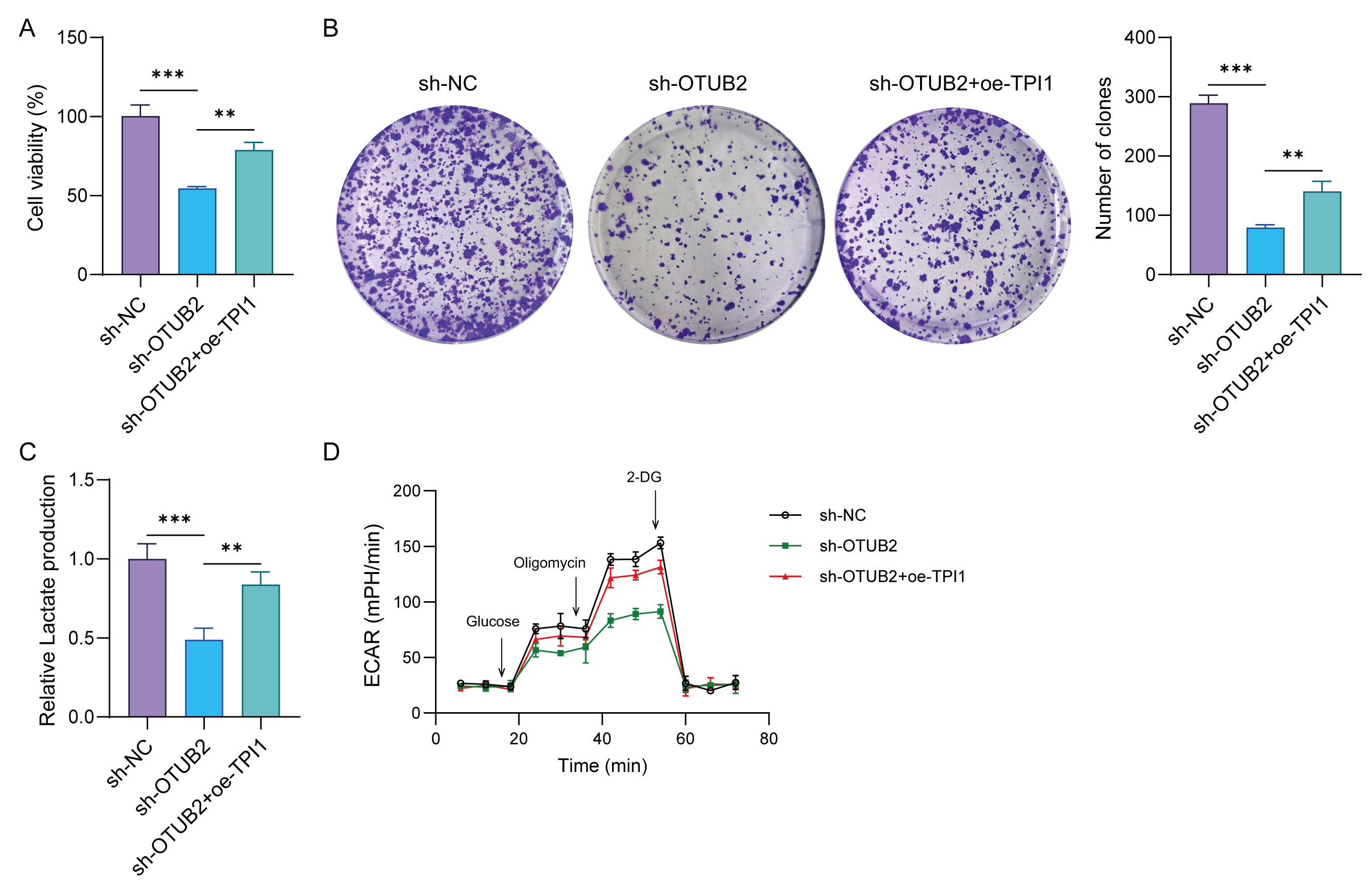

Supplement: Supplementary file 7 — Supplementary Material 7. OTUB2 silencing reduces the proliferation and glycolysis in MDA-MB-468 cells via regulation of TPI1. MDA-MB-468 cells were infected with sh-OTUB2 or sh-NC; or co-infected with sh-OTUB2 and oe-TPI1. (A-B) CCK-8 and colony-forming assays were employed to assess cell proliferation. (C-D) Lactate production and the ECAR of cells were detected. n = 3 per group; Results are expressed as mean ± SD. Statistical analyses were analyzed using an one-way ANOVA followed by Tukey’s multiple-comparisons test. **p < 0.01; ***p < 0.001 [file 13058_2026_2260_MOESM7_ESM.tif]
